# IDO-Mediated Immune and Metabolic Dysregulation in Schwann Cells Exposed to *Mycobacterium leprae*

**DOI:** 10.3390/cells14191550

**Published:** 2025-10-03

**Authors:** Atta Ur Rahman, Raíssa Couto Santana, Mylena Masseno de Pinho Pereira, Claudia Luciana dos Santos Moura, Débora Santos da Silva, Otto Castro Araujo, Thyago Leal-Calvo, Isabela Espasandin, Tatiana Pereira da Silva, Euzenir Nunes Sarno, Bruno Jorge de Andrade Silva, Rubem Sadok Figueiredo Menna-Barreto, Márcia Maria Jardim, Cristiana Santos de Macedo, Flávio Alves Lara, Roberta Olmo Pinheiro

**Affiliations:** 1 Leprosy Laboratory, Oswaldo Cruz Institute, Oswaldo Cruz Foundation, Rio de Janeiro 21040-361, Brazil; atta25394@gmail.com (A.U.R.); raissa.couto@hotmail.com.br (R.C.S.); mylena.masseno@gmail.com (M.M.d.P.P.); claudia.lucianasm2004@gmail.com (C.L.d.S.M.); thyagoleal@yahoo.com (T.L.-C.); isabela.espasandin@gmail.com (I.E.); euzenir@fiocruz.br (E.N.S.); brunojas85@ucla.edu (B.J.d.A.S.); jardim.mm@gmail.com (M.M.J.); 2 Cellular Microbiology Laboratory, Oswaldo Cruz Institute, Oswaldo Cruz Foundation, Rio de Janeiro 21040-361, Brazil; dradeborasantos85@gmail.com (D.S.d.S.); ottocastron@gmail.com (O.C.A.); cristiana.macedo@fiocruz.br (C.S.d.M.); falara@ioc.fiocruz.br (F.A.L.); 3 Mycobacteria Network, Inova-IOC Program, Oswaldo Cruz Institute, Oswaldo Cruz Foundation, Rio de Janeiro 21040-361, Brazil; 4 Innovative Genomics Institute, University of California, Berkeley, CA 94720, USA; 5 Cellular Biology Laboratory, Oswaldo Cruz Institute, Oswaldo Cruz Foundation, Rio de Janeiro 21040-361, Brazil; rubemb@ioc.fiocruz.br; 6 Post-Graduate Program in Neurology, Federal University of the State of Rio de Janeiro, Rio de Janeiro 21941-853, Brazil; 7 Department of Neurology, Pedro Ernesto University Hospital, Rio de Janeiro State University, Rio de Janeiro 20551-030, Brazil; 8 Rio de Janeiro Research Network on Neuroinflammation, Oswaldo Cruz Institute, Oswaldo Cruz Foundation, Rio de Janeiro 21040-361, Brazil; 9 National Institute of Science and Technology on Neuroimmunomodulation, Oswaldo Cruz Institute, Oswaldo Cruz Foundation, Rio de Janeiro 21040-361, Brazil; 10 Center for Technological Development in Health (CDTS), Oswaldo Cruz Foundation (FIOCRUZ), Rio de Janeiro 21040-361, Brazil

**Keywords:** *Mycobacterium leprae*, Schwann cells, Indoleamine 2,3 Dioxygenase (IDO), kynurenine pathway

## Abstract

Leprosy is a chronic infectious disease that targets the peripheral nervous system, leading to peripheral neuropathy. *Mycobacterium leprae* primarily infects Schwann cells, adipocytes, and macrophages, altering their metabolism and gene expression. This study investigates the metabolic interaction between *M. leprae* and Schwann cells, with a focus on indoleamine 2,3-dioxygenase (IDO), a key enzyme in tryptophan catabolism via the kynurenine pathway. We found that *M. leprae* induces IDO expression in Schwann cells, suggesting a role in immune modulation and neuropathy. Inhibition of IDO with 1-methyl-L-tryptophan (1-MT) reduced Schwann cell viability and metabolic activity in response to *M. leprae*. After 24 h of infection, *M. leprae* impaired mitochondrial membrane potential, although no significant changes in autophagy or mitochondrial ultrastructure were observed by electron microscopy. Interestingly, IDO1 inhibition upregulated the expression of antioxidant genes, including *GPX4*, *NFE2L2*, and *HMOX1*. In conclusion, these findings highlight a central role for IDO in shaping the metabolic and immunological response of Schwann cells to *M. leprae* infection. IDO induction contributes to immune regulation and cellular stress, while its inhibition disrupts cell viability and promotes antioxidant gene expression. These results position IDO as a potential therapeutic target for modulating host–pathogen interactions and mitigating nerve damage in leprosy.

## 1. Introduction

Leprosy, or Hansen’s disease, is a chronic granulomatous infectious condition caused by *Mycobacterium leprae* and *Mycobacterium lepromatosis*. It is characterized by slow progression and primarily affects the mucosal surfaces of the respiratory tract, skin, eyes, and peripheral nervous system (PNS) [1,2] Schwann cells, the main glial cells of the PNS, are key targets of *M. leprae* and play a critical role in maintaining nerve integrity and facilitating repair. Infection of the Schwann cells can significantly impair their metabolic activity and viability, ultimately leading to nerve dysfunction and peripheral neuropathy [3,4].

The invasion of peripheral nerves by *M. leprae* has long been recognized as a major contributor to neural damage in leprosy, regardless of the host’s immunological response or the presence of leprosy reactions [5]. *M. leprae* modulates Schwann cell function by inducing lipid droplet formation, which facilitates bacterial survival through inhibition of phagosome maturation and disruption of host energy metabolism [6]. Additionally, the bacilli can reprogram Schwann cells into progenitor-like states, impairing their roles in nerve maintenance and regeneration [7].

One of the key mechanisms implicated in immune modulation in leprosy is the induction of Indoleamine 2,3-dioxygenase (IDO). The indoleamine 2,3-dioxygenase enzyme exists in two isoforms, IDO1 and IDO2, which catalyze the initial and rate-limiting step of tryptophan degradation along the kynurenine pathway (KP); while IDO1 is well characterized and broadly expressed in immune and non-immune cells, IDO2 shares partial functional overlap but displays more restricted tissue distribution and lower enzymatic activity [8].

*M. leprae* induces IDO1 expression in human monocytes, with elevated levels observed in patients with the lepromatous (LL) form of leprosy compared to the tuberculoid (TT) form [9,10]. Patients with the LL form may develop a condition known as silent neuropathy, characterized by progressive nerve damage in the absence of overt inflammation or clinical symptoms [11,12,13]. Upregulation of IDO in infected tissues, particularly in macrophages and dendritic cells, promotes bacterial persistence by shaping an immunosuppressive environment [14,15,16]. Furthermore, IDO influences the balance between effector and regulatory T cells, contributing to immune dysregulation in leprosy [9,16].

Cells isolated from lepromatous lesions exhibited a CD68^+^IDO^+^ phenotype, and higher soluble IL-10 levels in sera positively correlated with IDO activity. In vitro, stimulation of healthy monocytes with increasing *M. leprae* concentrations induced IDO expression alongside a regulatory cytokine profile including IL-10, TNF, and TGF-β. Importantly, IL-10 blockades significantly reduced *M. leprae*-induced IDO expression, highlighting IL-10 as a key mediator of IDO induction. These findings indicate that IL-10 plays a central role in modulating IDO expression and function, contributing to the establishment of a regulatory environment that favors bacterial persistence [10].

At the tuberculoid end of the disease spectrum, where IFN-γ expression is dominant, IDO-1 is likely induced primarily by this cytokine and may be linked to microbicidal activity [17], suggesting that in leprosy, IDO-1 can play a dual role, functioning either as a tolerogenic or microbicidal mediator depending on the local environment and the cytokines driving its expression. In leprosy skin lesions, besides intact bacilli, mycobacterial components from the cell membrane and cytosol released following bacterial death—a process driven by both the host immune response and treatment—are expected to trigger immune responses [18]. IDO plays dual roles in immune regulation and neurotoxicity by producing several biologically active metabolites. Among these, kynurenine (KYN) serves as a central intermediate, which can be further converted into either neuroprotective or neurotoxic compounds [19,20,21].

Disruption of tryptophan metabolism through KP activation has been extensively studied in central nervous system (CNS) disorders, but its role in peripheral neuropathy, particularly in the context of leprosy, remains poorly understood.

The mechanisms underlying the progression of nerve damage in this immunosuppressive environment remain unclear. It is not yet known whether metabolic modulation of Schwann cells by the bacillus itself may disrupt this suppressive milieu, thereby permitting nerve damage to occur. In this context, IDO1 emerges as a potential mediator, given its ability to generate metabolites capable of directly affecting the viability and functionality of neural cells [19,20,21]. Given that Schwann cells are the primary targets of *M. leprae* in the peripheral nervous system, assessing IDO1 activity in these cells may provide crucial insights into the local immunometabolic mechanisms contributing to nerve damage in leprosy. Despite these insights, the role of IDO in non-immune cells such as Schwann cells during *M. leprae* infection is not well characterized. Clarifying the impact of IDO activity on Schwann cell metabolism, viability, and the local inflammatory environment could reveal new mechanisms involved in leprosy-associated neuropathy. Targeting IDO and the kynurenine pathway may offer novel therapeutic strategies to mitigate nerve damage, restore immune homeostasis, and improve clinical outcomes in leprosy. The present study aims to explore the role of IDO1 in Schwann cell responses to *M. leprae*, with a focus on metabolic activity.

## 2. Materials and Methods

### 2.1. Cell Culture and Stimulation

For in vitro assays, the human Schwann cell line ST88-14, derived from a malignant peripheral nerve sheath tumor in a patient with neurofibromatosis type 1, was used (provided by Dr. J.A. Fletcher, Dana Farber Cancer Institute, Boston, MA, USA). Prior to experiments, cells were cultured in RPMI medium (Gibco BRL, Grand Island, NY, USA) supplemented with 10% fetal bovine serum (FBS, Gibco Invitrogen, Waltham, MA, USA), 2 mM L-glutamine (Gibco Invitrogen, USA), and 100 U/mL penicillin plus 100 μg/mL streptomycin (Pen/Strep, Gibco Invitrogen, USA). Additionally, routine screening of ST88-14 cells line was conducted for any mycoplasma contamination through polymerase chain reaction (PCR) to ensure the cell culture reliability and integrity. All the experiments were performed after the third passage of the cell culture to ensure consistent cellular characteristics according to the laboratory protocol. Unless otherwise specified, cells were incubated with irradiated *M. leprae* at 10, 50, or 100 μg/mL, with or without 1 mM 1-methyl-L-tryptophan (1-MT—cat. Number: 447439 Sigma-Aldrich, St. Louis, MO, USA), an IDO1 inhibitor, for 24 h at 37 °C in a 5% CO_2_ atmosphere. Irradiated *M. leprae* (strain NHDP, gamma-irradiated whole cells, lyophilized, NR-19326) was obtained from BEI Resources, NIAID, NIH, Manassas, VA, USA), and stored at −20 °C in 1 mg/mL aliquots.

### 2.2. Microarray Analysis

Microarray analysis was used to evaluate gene expressions related to the kynurenine pathway in Schwann cells. Data from infected primary human Schwann cells (*M. leprae* Thai-53, MOI 100:1, 24 or 48 h at 33 °C; NCBI Gene Expression Omnibus accession number GSE40950) were analyzed using RStudio (v3.4.1) and the Pretty Heatmap package (v1.0.12). Heatmaps were generated from log2-transformed, normalized expression values. R computer source code (v. 3.4.1) and data used in the analyzes are readily available at https://doi.org/10.5281/zenodo.3840319.

### 2.3. Flow Cytometry Assay

IDO1 expression was quantified in ST88-14 Schwann cells using flow cytometry. A total of 1 × 10^5^ cells were seeded in 6-well plates and treated as indicated. After treatment, cells were harvested using 0.25% trypsin/1 mM EDTA, washed with FACS buffer (PBS + 0.1% BSA), and fixed with 4% paraformaldehyde for 30 min at 4 °C. Permeabilization was performed using PBS containing 0.1% saponin and 1% BSA. Cells were then blocked with 2% normal goat serum for 15 min at room temperature. Intracellular staining was conducted using a PE-conjugated anti-IDO1 antibody (R&D Systems, Minneapolis, MN, USA; 1:50 dilution, cat. IC6030P) for 30 min at room temperature in the dark. A PE-conjugated isotype control antibody (R&D Systems, cat. IC002P) was used to assess non-specific staining. Samples were filtered through 40 μm cell strainers to minimize cell aggregates and analyzed on a BD FACSCalibur flow cytometer (BD Biosciences, San Jose, CA, USA). Data were acquired using standard instrument settings that collect only pulse area (integrated signal) parameters. As FACSCalibur does not provide pulse height or width data, conventional singlet gating (e.g., FSC-A vs. FSC-H) could not be performed. To reduce the inclusion of doublets or aggregates, we applied tight forward scatter (FSC) vs. side scatter (SSC) gating to select the main cell population. A minimum of 10,000 events was collected per sample. Data were analyzed using FlowJo software v10 (BD Biosciences, Ashland, OR, USA).

### 2.4. High-Performance Liquid Chromatography (HPLC)

HPLC was used to quantify kynurenine (Kyn) and tryptophan (Tryp) levels in supernatants. Samples of supernatants were mixed with 5 μL of 10 mM tyrosine and 25 μL trichloroacetic acid in 400 μL supernatant, vortexed, and centrifuged at 14,000× *g* for 10 min. The supernatant was injected into a Shim-pack CLC-ODS(M)^®^ C18 column using a Shimadzu LC-20AT system (Shimadzu Corporation, Kyoto, Japan). The mobile phase consisted of 15 mM sodium acetate buffer and acetonitrile with 0.1% trifluoroacetic acid. Absorbance was read at 365 nm (Kyn) and 285 nm (Tryp), with tyrosine as the internal standard. IDO1 activity was expressed as the Kyn/Tryp peak area ratio [22].

### 2.5. Evaluation of Schwann Cell Metabolic Activity

Schwann cell line ST88-14 (8000/well) were plated in 96-well plates and treated with irradiated *M. leprae*, 1-MT, or their combinations for 24 h. Metabolic activity was assessed using the MTT assay (Thermo Fisher). MTT solution (10 μL, 5 mg/mL) was added to each well; reduced formazan crystals were solubilized in DMSO and read at 590 nm using a SpectraMax 190 spectrophotometer (San Jose, CA, USA. Mitochondrial membrane potential (Δψm) was assessed using 1 nM TMRM (Sigma Aldrich–Cat. T5428, Burlington, MA, USA) staining. After 24 h incubation with irradiated *M. leprae* (with or without 1-MT), Schwann cells (10,000/well) were stained for 20 min and imaged using the Cytell™ Cell Imaging System (GE Healthcare, Chicago, IL, USA), measuring changes in mitochondrial polarization [3]. The results were shown by the ratio of the TMRM signal to the same signal from a culture preincubated with the proton ionophore CCCP (15 μM) (Sigma) for 10 min.

### 2.6. Cell Viability Assay

Cell viability was evaluated using the Differential Nuclear Staining (DNS) assay. Schwann cell line ST88-14 (10,000/well) were stained with Hoechst 33,342 (Thermo Fisher Scientific, Waltham, MA, USA) for 30 min and Propidium Iodide (PI) (Life Technologies Corporation, Eugene, OR, USA) for 5 min. Hoechst stains all nuclei, while PI selectively stains dead cells. Fluorescence was quantified using the Cytell system [23].

### 2.7. Transmission Electron Microscopy (TEM) Analysis

Schwann cell line ST88-14 (5 × 10^5^/well) were incubated with irradiated *M. leprae* (50 μg/mL), with or without 1-MT, for 24 h. Cells were fixed with 2.5% glutaraldehyde in 0.1 M cacodylate buffer, post-fixed with 1% OsO_4_, dehydrated in graded acetone, and embedded in PolyBed 812 resin. Ultrathin sections were stained with uranyl acetate and lead citrate and imaged with a JEM1011 (Jeol, Akishima, Tokyo, Japan) or HT7800 (Hitachi, Tokyo, Japan) microscope at the Electron Microscopy Platform, Instituto Oswaldo Cruz (Fiocruz).

### 2.8. Western Blotting

To assess autophagy, LC3B expression was evaluated. Schwann cell line ST88-14 (4 × 10^5^/well) were lysed after 24 h incubation. Proteins (20 μg/sample) were separated by 12% SDS-PAGE, transferred to nitrocellulose membranes, and blocked with 5% BSA or milk. Membranes were incubated overnight at 4 °C with anti-LC3B (1:1000) and anti-Tubulin (1:4000), followed by HRP-conjugated secondary antibodies (1:1000). Detection was performed using ECL and visualized on an iBright CL750 system. Densitometric analysis was performed in ImageJ 1.53t.

### 2.9. RNA Extraction and RT-qPCR

Total RNA was extracted with TRIzol (Invitrogen) following the manufacturer’s instructions, treated with DNase, and quantified using NanoDrop (ThermoFisher Scientific). cDNA synthesis (1500 ng RNA) was performed according to the manufacturer instruction with the Superscript III kit (Invitrogen). RT-qPCR was carried out with SYBR Green Master Mix (Applied Biosystems) on a StepOnePlus™ system using a Schwann cell-targeted primer array (GenOne, Brazil). *SQSTM1* was evaluated and *GAPDH* and *RPL13A* served as reference genes for normalization. Data was analyzed with LinRegPCR (2021.2.0.0). While for genes *GPX4* (Hs00989766_g1), *NFE2L2* (Hs00232352_m1), *NCOA4* (Hs00428331_g1), *HMOX* (Hs01110250_m1), and *ACSL4* (Hs00244871_m1), TaqMan assays (Thermo Fisher Scientific) with FAM probe were used for expression analysis. The TaqMan data was analyzed using 2^−ΔΔCT^ method and normalized using the *ACTB* (Hs01060665_g1) and *GAPDH* (Hs99999905_m1) as internal control.

### 2.10. Statistical Analysis

All data were analyzed using GraphPad Prism version 8.0.2 (GraphPad Software, San Diego, CA, USA). Data distribution was assessed for normality using the Shapiro–Wilk test, and homogeneity of variances was evaluated using Levene’s test. For datasets that met assumptions of normality and equal variance, parametric tests (e.g., one-way ANOVA followed by Tukey’s multiple comparison test) were applied. For non-normally distributed data or data with unequal variances, non-parametric tests were used, including the Kruskal–Wallis test followed by Dunn’s multiple comparison test.

Results are presented as mean ± SD, as indicated in the figure legends. The number of independent biological replicates (n) is reported in each figure panel. Statistical significance was defined as *p* ≤ 0.05. Where possible, exact *p* values and effect sizes are provided in the figure legends. Multiple comparisons were adjusted using appropriate post hoc tests, as specified above.

## 3. Results

### 3.1. M. leprae Influences Tryptophan Catabolism and Mitochondrial Function in Human Schwann Cells

**Figure 1:**
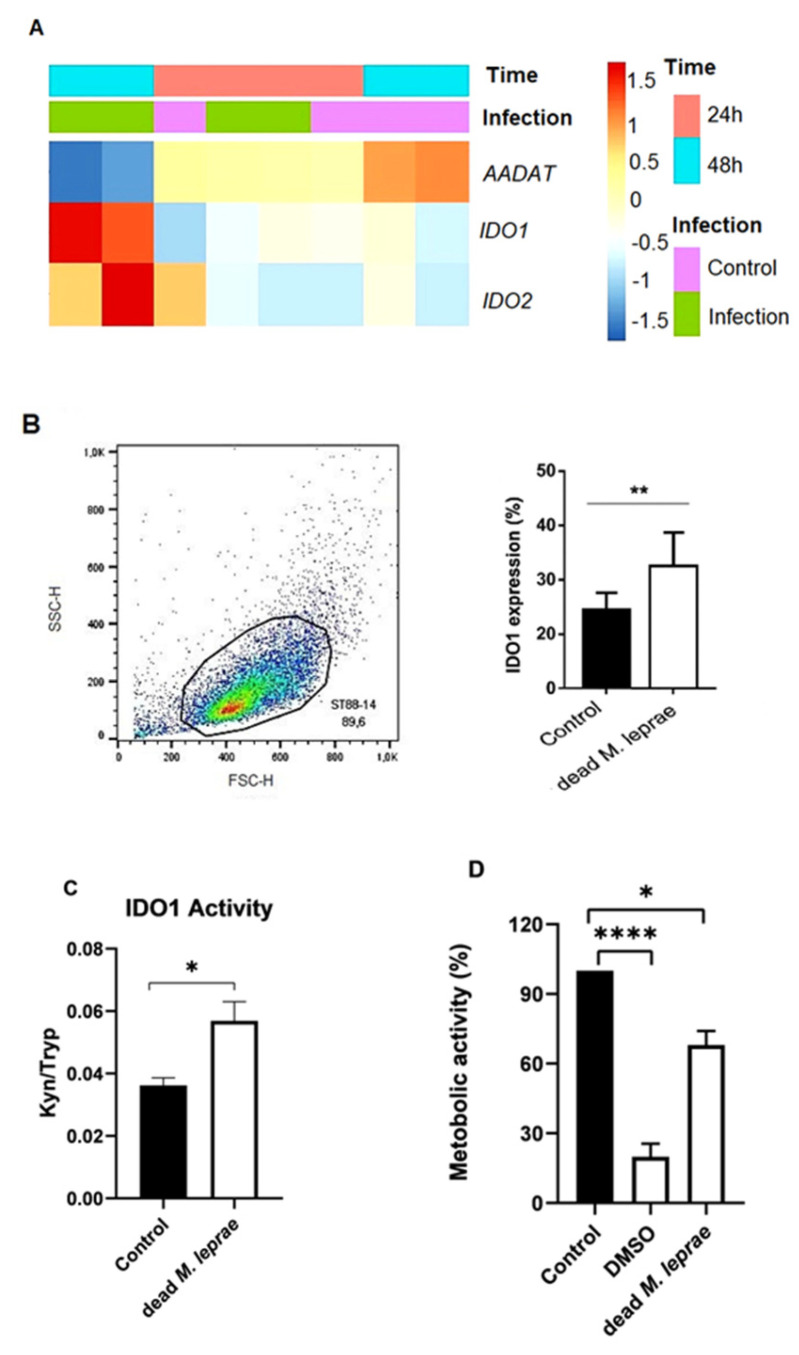
*M. leprae* differentially modulates IDO expression and Schwann cell metabolism. (**A**) Heatmap represents the IDO and *AADAT* genes expression in primary Schwann cells infected (in green) or not (in purple) with *M. leprae* (for 24 h (in pink) and 48 h (in blue). (**B**) Gate strategy in the cell’s population through the FSC-H and SSC-H and percentage of IDO1 positive cells (%). The FACScalibur flow cytometer was used for the acquisition and FlowJo software was used for the analysis. Statistical analysis of n = 6 independent experiment was performed by using the unpaired *t*-test. The data are represented as mean ± SD. (**C**) The figure represents the HPLC for IDO1 activity of the culture supernatant by kyn/tryp ratio. Statistical analysis of n = 5 independent experiments were performed using the unpaired *t*-test (*p* = 0.0253). The data was represented as a mean with SEM. (**D**) The metabolic activity of Schwann cells incubated with irradiated *M. leprae*. Statistical analysis of n = 5 independent experiments were performed by Kruskal–Wallis non-parametric followed by Dunn’s multiple comparisons test (*p* ≤ 0.0001). The data was represented as mean ± SD. Where **** means *p* < 0.0001, ** means *p* < 0.005 and * means *p* < 0.05.

Global gene expression analysis using microarrays was performed to identify genes regulated in Schwann cells after 24 h of infection with *M. leprae*. In a previous study from our group, we analyzed Schwann cells infected with a multiplicity of infection (MOI) of 100:1 [24]. For the present study, a bioinformatic reanalysis was conducted to evaluate whether *M. leprae* could modulate the expression of IDO or other genes related to the kynurenine pathway in Schwann cells. As shown in Figure 1A, *M. leprae* increased the expression of both IDO1 and IDO2, while reducing the expression of *AADAT*, the gene encoding kynurenine aminotransferase II. Both IDO1 and IDO2 catalyze the first and rate-limiting step in the catabolism of tryptophan to kynurenines; however, previous studies have shown that IDO2 has significantly weaker catalytic activity compared to IDO1 [25,26,27].

Since our earlier data demonstrated that *M. leprae* can induce IDO1 expression in monocytes [9], we investigated whether the bacillus could also induce IDO1 in the human Schwann cell line ST88-14. *M. leprae* (50 µg/mL) significantly increased both IDO1 expression and enzymatic activity in ST88-14 cells (Figure 1B,C). IDO1 catalyzes the conversion of L-tryptophan into kynurenine, thereby activating the kynurenine pathway.

To assess the metabolic effects of *M. leprae*, we performed an MTT assay, which measures cellular metabolic activity through the reduction in the yellow tetrazolium salt into purple formazan crystals by mitochondrial enzymes in viable cells. As shown in Figure 1D, irradiated (dead) *M. leprae* significantly reduced the metabolic activity of ST88-14 cells, while DMSO (10%) was used as a positive control for cell death.

### 3.2. IDO Inhibition Reduces Metabolic Activity and Viability in ST88-14 Cells Independent of M. leprae

**Figure 2:**
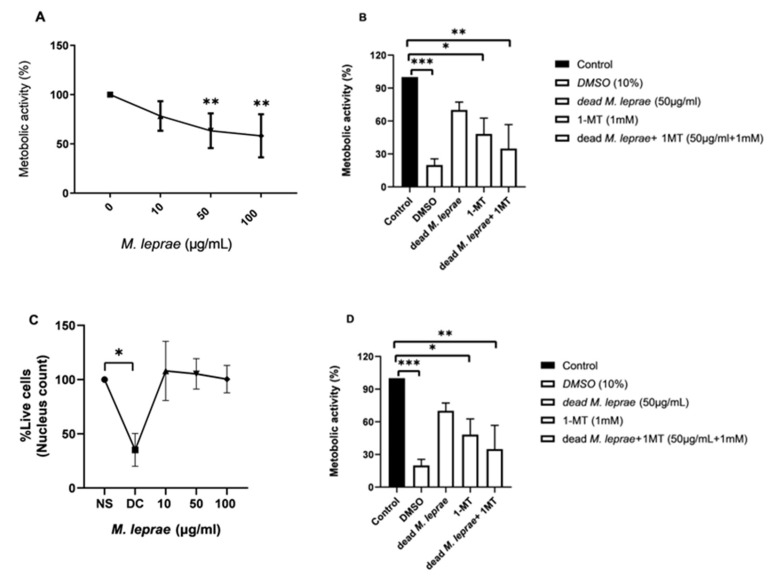
Differential Impact of *M. leprae* and 1-MT on Schwann Cell Metabolic Activity and Viability.(**A**,**B**) Metabolic activity of Schwann cells treated with irradiated (dead) *M. leprae*, 1-MT, or both in combination. ((**A**): *p* = 0.023; (**B**): *p* = 0.0003).(**C**,**D**) Viability of Schwann cells exposed to M. leprae, 1-MT, or their combination ((**C**): *p* = 0.0155; (**D**): *p* = 0.0003). Statistical analysis was performed on five independent experiments (n = 5) using Kruskal–Wallis non-parametric test followed by Dunn’s multiple comparisons test. Data are presented as mean ± SD. Asterisks indicate statistical significance: *** *p* < 0.001, ** *p* < 0.005, * *p* < 0.05.

To investigate the role of IDO in Schwann cell metabolism, we pretreated ST88-14 cells with the IDO inhibitor 1-MT prior to stimulation with *M. leprae*. Irradiated (dead) *M. leprae* reduced cellular metabolic activity in a dose-dependent manner (Figure 2A). Treatment with 1-MT significantly decreased metabolic activity, regardless of the presence of dead bacilli (Figure 2B). Compared to control, dead *M. leprae* alone did not significantly affect Schwann cell viability, whereas 1-MT, either alone or in combination with bacilli, led to a significant reduction in cell viability (Figure 2C,D).

### 3.3. Functional Consequences of IDO Blockade: Impaired Autophagy, Preserved Mitochondria, and Activated Antioxidant Pathway

**Figure 3:**
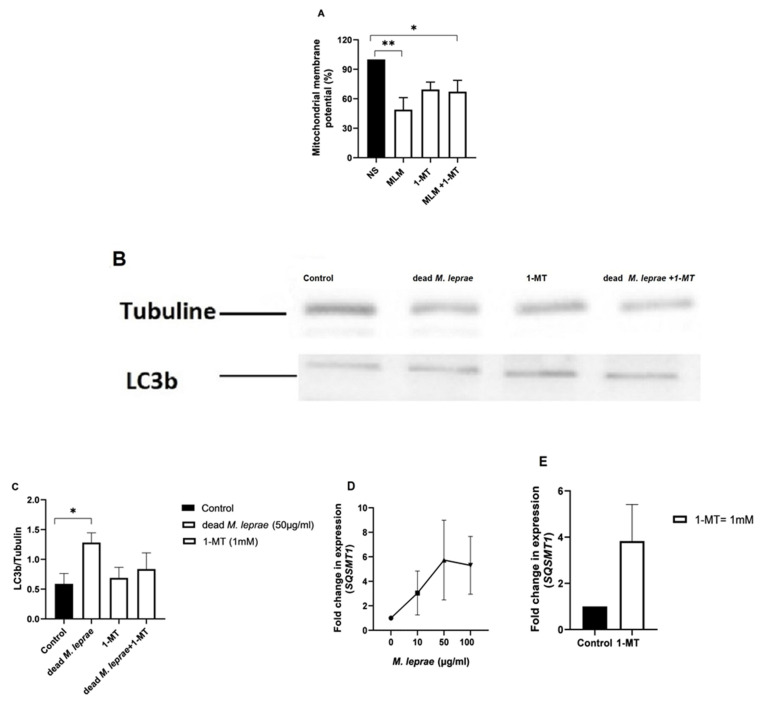
Autophagy Modulation in Schwann Cells Exposed to *M. leprae* and 1-MT. (**A**) Mitochondrial membrane potential of Schwann cells treated with irradiated (dead) *M. leprae* and 1-MT (*p* = 0.0021). Results were analyzed using Kruskal–Wallis non-parametric test, followed by Dunn’s multiple comparisons test. Data are presented as mean ± SD from n = 4 independent biological replicates. (**B**,**C**) Representative Western blot bands and relative expression of LC3-II normalized to Tubulin. Dead *M. leprae* significantly increased LC3-II protein levels (*p* = 0.0428). Statistical analysis was performed using Kruskal–Wallis with Dunn’s test; data are presented as mean ± SD from n = 4 independent experiments. (**D**) Gene expression analysis of *SQSTM1* in cells treated with dead *M. leprae* (n = 3, *p* = 0.0642), analyzed using Kruskal–Wallis followed by Dunn’s test. Data are shown as mean ± SD. (**E**) *SQSTM1* expression in cells treated with the IDO inhibitor 1-MT. Results were analyzed using an unpaired *t*-test (*p* = 0.1470) and are presented as mean ± SD from n = 3 independent replicates. Asterisks indicate statistical significance: ** *p* < 0.005; * *p* < 0.05.

Cells were treated with *M. leprae* and IDO inhibitor to evaluate their impact on mitochondrial function (ΔΨm), autophagy, and the cells’ antioxidant defense pathway. A decrease in mitochondria activity (ΔΨm) was observed in cells treated with *M. leprae* and in combination with 1-MT (Figure 3A). Furthermore, Western blotting analysis showed an increase in LC3-II expression after exposure to dead *M. leprae*, but not with 1-MT (Figure 3B,C). Additionally, *M. leprae* altered p62 expression in a concentration-dependent manner, and IDO inhibition comparatively increased p62 levels in ST88-14 cells (Figure 3D,E). p62 (also known as *SQSTM1*) is a multifunctional adaptor protein involved in selective autophagy. It binds ubiquitinated proteins and directs them to the lysosome via interaction with LC3. An increase in p62 typically suggests impaired autophagic flux.

**Figure 4:**
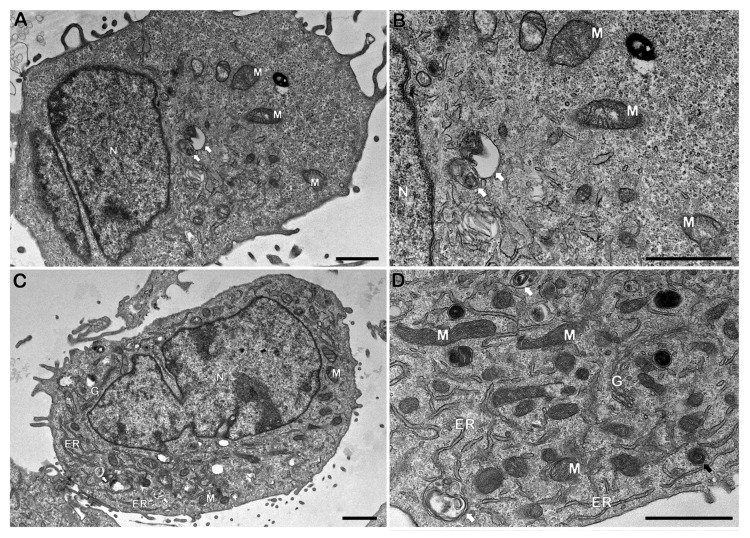
Ultrastructural analysis of Schwann cells incubated with irradiated *M. leprae* for 24 h. (**A**,**B**) Control cells (without *M. leprae*) showing typical morphology of nucleus (N) and mitochondria (M), as well as few autophagosomes (white arrows) can also be observed. In cells exposed to dead *M. leprae* (**C**,**D**), endoplasmic reticulum (ER) profiles, Golgi (G), mitochondria (M), autophagosomes (white arrows) and concentric membrane structures (black arrows) in the cytosol were commonly detected. Bars: 1 µm.

To further investigate whether bacteria and IDO inhibition may bring structural alterations in Schwann cell line. Analysis of ultrastructural changes by electron microscopy (Figure 4) did not reveal significant differences in autophagic morphology in Schwann cells incubated with irradiated *M. leprae* for 24 h when compared to control cells.

**Figure 5:**
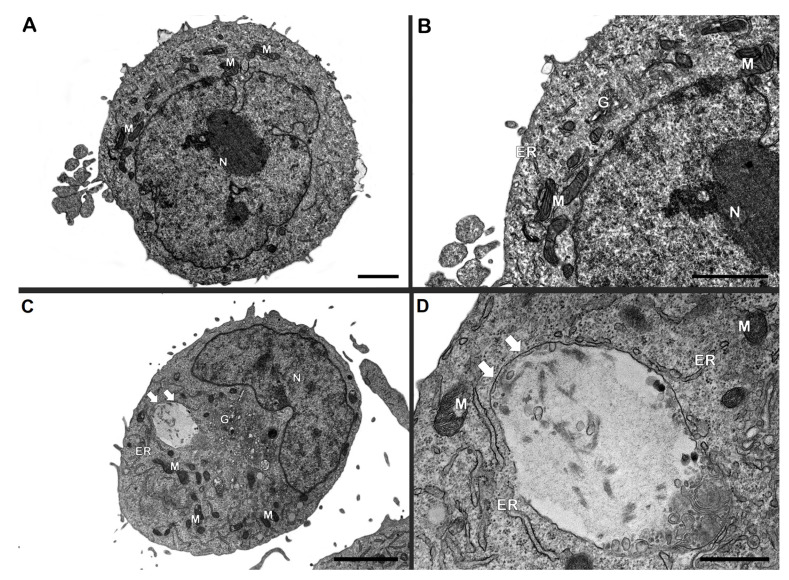
Ultrastructural analysis of Schwann cells incubated with *M. leprae* and treated with IDO inhibitor for 24 h. (**A**,**B**) Cells treated with the IDO inhibitor (1-MT) presenting normal morphological aspects of nucleus (N) and mitochondria (M) and Golgi (G). Cells incubated with dead bacilli+1-MT (**C**,**D**) showed no significant differences to control, with typical characteristics of endoplasmic reticulum (ER) profiles, Golgi (G), mitochondria (M) and autophagosomes (white arrow). Bars: 2 µm; Bar inset D: 0.5 µm.

In the time observed, mitochondria showed no major ultrastructural changes in cells exposed to *M. leprae* (Figure 4C,D). Additionally, the cells were also treated with IDO inhibitor (1-MT) alone or in co-incubation with dead *M. leprae,* and no typical alteration was observed in ultrastructural study (Figure 5A–D). After *M. leprae* exposure and IDO inhibition, mitochondrial morphology and the number of autophagosomes remained consistent across all experimental groups.

**Figure 6:**
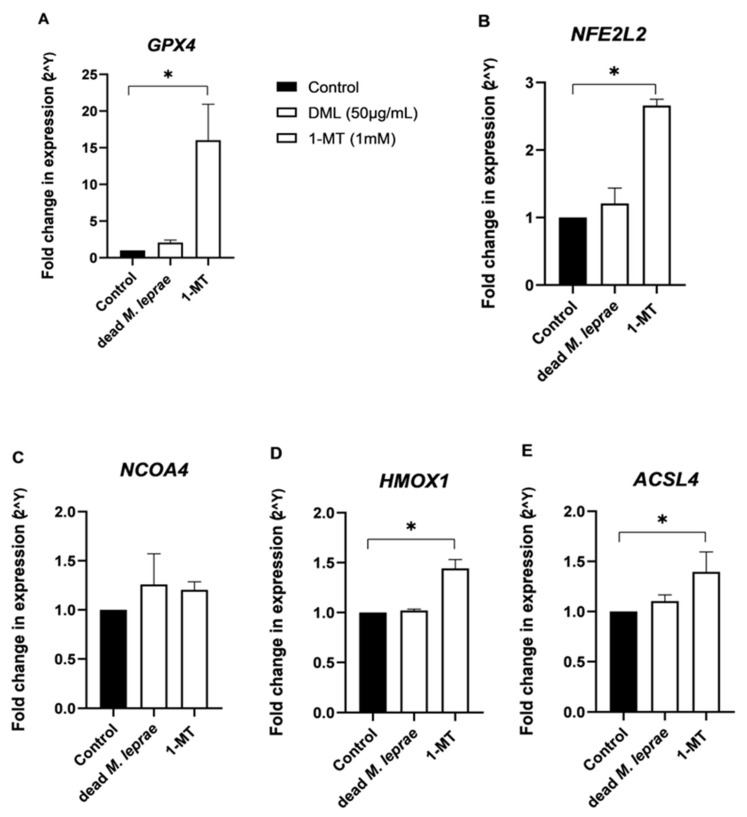
*M. leprae* and 1-MT Trigger Oxidative Stress in Schwann Cells. (**A**,**B**) Upregulation of *GPX4* and *NFE2L2* was found in response to both stimuli, with significant induction in 1-MT–treated Schwann cells ((**A**): *p* = 0.0286, (**B**): *p* = 0.0286). (**C**) The expressions of *NCOA4* were not affected (*p* = 0.0572), while (**D**,**E**) *HMOX1* and *ACSL4* were significantly upregulated in cells exposed to 1-MT ((**D**): *p* = 0.0286, (**E**): *p* = 0.0286). Statistical analysis of n = 3 independent experiments were performed by Kruskal–Wallis non-parametric followed by Dunn’s multiple comparisons test. The data was represented as mean ± SD. where * means *p* < 0.05.

Although ultrastructural damage was not observed within the evaluation window, the possibility of oxidative stress cannot be ruled out. Analysis of cell cultures over longer periods led to greater detachment of cells from the culture plate and greater loss of cell viability, with damage to mitochondrial activity, which was more prominent at 48 h and 72 h. This oxidative stress may activate the *NFE2L2* → *GPX4/HMOX1* antioxidant axis as a protective mechanism against ROS and lipid peroxidation. As shown in Figure 6, IDO inhibition increased the expression of *GPX4*, *NFE2L2*, and *HMOX1*. These findings suggest that IDO may play a suppressive role in the activation of the *NFE2L2* → *GPX4/HMOX1* antioxidant pathway, and that its inhibition enhances the cellular antioxidant response to oxidative stress.

IDO inhibition by 1-MT significantly impaired Schwann cell metabolism and viability, independent of *M. leprae* infection, highlighting a central role for IDO in maintaining Schwann cell homeostasis and suggesting its potential as a therapeutic target to mitigate nerve damage in leprosy.

## 4. Discussion

Despite significant advances in molecular and cellular techniques in recent years—enabling a deeper understanding of the immunopathogenic mechanisms underlying various infectious diseases—many aspects of leprosy pathogenesis remain poorly understood. A major obstacle to progress in this field is the lack of a robust experimental model. Moreover, *Mycobacterium leprae*, the primary etiological agent, cannot be cultured in conventional laboratory media, further limiting research. One of the most pressing challenges is unraveling the mechanisms of leprosy-associated neuropathy.

It is well established that *M. leprae* infects macrophages and adipocytes in the dermis, as well as Schwann cells in peripheral nerves [28]. Nerve damage may result from both the direct interaction of the bacillus with infected cells and from inflammatory mediators produced by immune infiltrates. Although multidrug therapy (MDT) remains a cornerstone for controlling disease transmission, it is not effective in preventing or managing nerve damage. Therefore, elucidating the immunopathogenic mechanisms involved in neural injury is critical for the development of adjunctive therapies aimed at reducing both infection and tissue damage.

In this study, we demonstrate for the first time that *M. leprae* can modulate indoleamine 2,3-dioxygenase (IDO1) expression and activity in Schwann cells. Our results show a significant increase in IDO1 expression in *M. leprae*-stimulated ST88-14 Schwann cells compared to controls. Notably, primary Schwann cells also exhibited elevated IDO1 expression, supporting a potential link between IDO1 activity and nerve damage. While these results were derived from distinct experimental models—ST88-14 cells in vitro and primary Schwann cells in the GSE40950 transcriptomic dataset—we observed consistent upregulation of IDO1 across both, reinforcing the robustness of the finding.

In the inflammatory milieu typical of leprosy neuropathy, bacillary death is common, and antigenic stimulation of resident cells occurs through both viable and dead bacilli. Thus, the immune response induced by irradiated *M. leprae* in vitro likely mimics key antigenic stimuli present in infected tissues. This supports the notion that, at least with regard to IDO1 modulation, the use of irradiated bacilli serves as a valid and practical experimental surrogate. Despite differences in cell type, temperature, and bacterial viability, the convergence of findings across models underscores the biological relevance of IDO1 induction in Schwann cell responses to *M. leprae*.

In the present study, we employed irradiated, non-viable *M. leprae*, which cannot actively transcribe mRNA and therefore does not fully engage pathways dependent on live bacilli, such as type I IFN induction via STING/TBK1/IRF3 signaling [29]. It may represent a limitation when considering responses that require transcriptionally active bacteria. However, in the context of IDO1 induction and metabolic modulation in Schwann cells, the contribution of IL-10, a downstream gene of type I IFN pathway, appears as pivotal as type I IFNs in shaping the regulatory response.

IL-10 produced by Schwann cells or surrounding immune cells can robustly induce and sustain IDO expression, promoting a tolerogenic and immunomodulatory environment even in the absence of live bacilli. Therefore, while the use of irradiated *M. leprae* does not capture the full spectrum of type I IFN–dependent responses, it remains a valid and informative model for interrogating pathways where IL-10–driven regulation of IDO is central, reflecting a physiologically relevant component of the inflammatory milieu observed in leprosy lesions. Although we recognize the inherent limitations of using non-viable bacilli, this approach allows mechanistic insights into IDO-mediated metabolic modulation, highlighting pathways that are operative in vivo and complementing studies that focus on live bacilli–dependent immune responses.

It is well documented that *M. leprae* disrupts Schwann cell metabolism [3]. Mitochondria—central organelles for energy production and immune signaling—regulate redox balance, apoptosis, inflammasome activation, and xenophagy. Schwann cells infected by *M. leprae* show a reduction in mitochondrial membrane potential (Δψm), increased glucose uptake, decreased mitochondrial activity, and reduced lactate production. These metabolic alterations reflect a shift toward glycolysis, accompanied by diminished pyruvate dehydrogenase activity and HIF-1α stabilization. These changes contribute to mitochondrial dysfunction, reduced β-oxidation of lipids, accumulation of lipid bodies, loss of axonal metabolic support, and ultimately nerve damage [3,30,31]. Our findings are consistent with these observations, demonstrating that *M. leprae* antigens impair Schwann cell metabolic function.

This mitochondrial dysfunction may reflect an adaptive cellular response aimed at limiting oxidative stress. Transcriptomic data have shown a downregulation of genes involved in the mitochondrial respiratory chain during *M. leprae* infection [24]. Mitochondria are major sources of reactive oxygen species (ROS), particularly superoxide anions produced at complexes I and III of the electron transport chain. These ROS can be converted into peroxynitrite and hydrogen peroxide, leading to damage of *M. leprae* lipids, proteins, and DNA [32,33].

In our study, Schwann cells exhibited decreased mitochondrial membrane potential (Δψm) and reduced mitochondrial activity following exposure to *M. leprae*, suggesting mitochondrial dysfunction in the absence of cell death. This is likely due to the activation of survival-promoting mechanisms.

Our results further show that inhibition of IDO1 using 1-methyl-L-tryptophan (1-MT) reduced Schwann cell viability and metabolic activity in the presence of *M. leprae*, as measured by the MTT assay. This finding suggests that IDO1 plays a supportive role in maintaining metabolic homeostasis during infection-induced stress. IDO1 catalyzes the first and rate-limiting step in the degradation of tryptophan via the kynurenine pathway, generating metabolites that influence redox balance, mitochondrial function, and NAD^+^ biosynthesis. Inhibition of this pathway may disrupt these processes, leading to impaired mitochondrial activity and increased cellular vulnerability. Since the MTT assay relies on the activity of mitochondrial dehydrogenases, it is sensitive to changes in metabolic and redox status. The observed reduction in MTT activity following IDO1 inhibition likely reflects compromised mitochondrial function. Additionally, reduced kynurenine levels may impair immunometabolic signaling, further contributing to the observed effects. These findings support the hypothesis that IDO1 contributes not only to immune modulation but also to the metabolic adaptation of Schwann cells during *M. leprae* infection.

Although this study did not comprehensively assess the autophagic process, we evaluated key markers, including LC3-II and p62, to obtain preliminary evidence of potential modulation. Our primary aim was not to fully characterize the autophagy pathway but rather to lay the groundwork for future studies exploring how *M. leprae* and IDO1 activity may influence autophagic flux in Schwann cells. Previous work from our group has shown that IDO1 may play either immunoregulatory or effector roles, depending on the inflammatory context. It is therefore plausible that the effect of IDO1 on autophagy is similarly context dependent.

To support the hypothesis that inflammatory mediators can modulate autophagy in Schwann cells, we showed that IFN-γ treatment increases autophagic flux in ST88-14 cells (Supplementary Materials Figure S1). This was visualized using monodansylcadaverine (MDC), a fluorescent marker of autophagic vacuoles, in combination with LysoTracker, confirming autolysosome formation under inflammatory stimulation. These findings reinforce the concept that Schwann cells have functional autophagy machinery that is responsive to inflammatory cues. Although we did not observe marked ultrastructural or molecular evidence of autophagy following *M. leprae* stimulation in this model, our results—together with the IFN-γ data and previous studies demonstrating *M. leprae*-induced myelinophagy in murine Schwann cells [34]—highlight the potential relevance of this pathway.

Future studies will focus on dissecting the mechanisms through which *M. leprae* and IDO1 influence autophagy, and how this interaction affects bacterial persistence and nerve damage in the context of leprosy neuropathy. Notably, our findings demonstrate that inhibition of IDO1 with 1-MT significantly reduces both metabolic activity and viability in human Schwann cells, independent of *M. leprae* infection. This underscores the central role of IDO1 in maintaining Schwann cell homeostasis. Through its contribution to NAD^+^ biosynthesis—an essential cofactor for mitochondrial energy metabolism—IDO1 activation appears critical for sustaining mitochondrial function and overall cell viability. Inhibition of this pathway may impair ATP production and lead to metabolic failure.

Furthermore, IDO1 inhibition led to the accumulation of p62 (*SQSTM1*), a key adaptor protein involved in autophagy. Under physiological conditions, p62 is continuously degraded through autophagic flux; its accumulation indicates impaired autophagy [35]. This suggests that IDO1 activity is necessary to maintain effective autophagic degradation in Schwann cells. Given the importance of autophagy in clearing damaged organelles and maintaining proteostasis [36,37], impaired autophagic flux could contribute to cellular stress and functional decline.

Previous studies from our group have shown that in skin lesion cells from multibacillary patients, there is an increase in IDO1 expression [9,10], accompanied by elevated levels of CD163—a scavenger receptor that binds haptoglobin-hemoglobin complexes and contributes to increased intracellular iron stores [10]. This accumulation of intracellular iron is associated with upregulation of the ferritin receptor and downregulation of ferroportin expression in macrophages from multibacillary patients [38].

Iron homeostasis plays a significant role in the regulation of ferroptosis, a form of iron-dependent lipid peroxidation that is negatively regulated by glutathione peroxidase 4 (*GPX4*) [39,40]. Elevated intracellular iron is a key factor in triggering this form of programmed cell death [41]. These observations support a link between increased IDO1 expression, altered iron metabolism, and enhanced susceptibility to ferroptosis in the context of leprosy.

Interestingly, despite evidence of reduced metabolic activity, mitochondrial morphology remained largely unaltered under electron microscopy. Although the MTT assay and mitochondrial membrane potential measurements showed a reduction in metabolic activity and mitochondrial function following stimulation with dead *M. leprae*, no significant alterations in mitochondrial or host cell morphology were observed at the 24 h time point.

This apparent discrepancy may be explained by the sensitivity and nature of the assays used. Functional assays like MTT and membrane potential assessments can detect early or subtle metabolic impairments that precede visible structural changes. Mitochondrial dysfunction can occur without immediate morphological damage, particularly during the early stages of stress or cellular adaptation [42,43]. Additionally, mitochondrial morphology is highly dynamic and regulated by fission–fusion processes, which may temporarily preserve organelle structure despite functional compromise. It is also possible that longer exposure times are required for ultrastructural alterations to become apparent, or that compensatory cellular mechanisms—such as antioxidant responses or autophagy—transiently maintain mitochondrial and cellular integrity under stress conditions induced by dead *M. leprae*.

*M. leprae* also induced the upregulation of the *NFE2L2 (NRF2)* → *GPX4/HMOX1* antioxidant response pathway. NRF2 is a master regulator of antioxidant defense; *GPX4* inhibits lipid peroxidation and ferroptosis, while *HMOX1* degrades heme into cytoprotective byproducts. Activation of this antioxidant axis suggests a compensatory response to oxidative stress triggered by mitochondrial dysfunction and impaired autophagy.

Taken together, our findings suggest that *M. leprae* modulates Schwann cell homeostasis through multiple, interconnected mechanisms involving immunometabolic reprogramming, mitochondrial dysfunction, autophagy modulation, and redox imbalance. The upregulation of IDO1 in both established and primary Schwann cell models reinforces its central role in cellular metabolism and immune regulation during infection. The observed reduction in cell viability and metabolic activity following IDO1 inhibition further underscores the enzyme’s importance for Schwann cell survival, even in the absence of bacilli. By contributing to NAD^+^ biosynthesis, IDO1 may support mitochondrial function and energy production, particularly under stress conditions. Moreover, its influence on autophagic flux—evidenced by p62 accumulation—indicates that IDO1 is also essential for maintaining cellular proteostasis, which is critical in metabolically active cells such as Schwann cells.

The mitochondrial and autophagic alterations observed following exposure to *M. leprae* antigens appear to reflect a complex adaptive response. Despite measurable declines in metabolic activity and mitochondrial membrane potential, the lack of marked ultrastructural changes at early time points suggests activation of compensatory mechanisms. The induction of the *NRF2–GPX4–HMOX1* antioxidant pathway further supports the idea that Schwann cells mount a cytoprotective response to infection-related oxidative stress. Importantly, this pathway also intersects with ferroptosis regulation, suggesting that *M. leprae* may actively modulate the redox environment of host cells to prevent cell death and promote persistence.

Together, these findings provide new insights into how metabolic and redox signaling pathways contribute to Schwann cell vulnerability and resilience during *M. leprae* infection and highlight IDO1 as a potential therapeutic target for mitigating nerve damage in leprosy.

## Data Availability

The original contributions presented in this study are included in the article/Supplementary Materials. Further inquiries can be directed to the corresponding author.

## Supplementary Materials

The following supporting information can be downloaded at https://www.mdpi.com/article/10.3390/cells14191550/s1, Figure S1: Interferon (IFN)γ induces autophagic flux in ST88-14 Schwann cells.
