# Supplementary material for: IDO-Mediated Immune and Metabolic Dysregulation in Schwann Cells Exposed to Mycobacterium leprae
Source: Cells. 2025 Oct 3;14(19):1550. doi: 10.3390/cells14191550 (PMC12524192; doi:10.3390/cells14191550)
Supplement: Supplementary file 1 [file cells-14-01550-s001.zip › cells-3824342-supplementary.pdf]

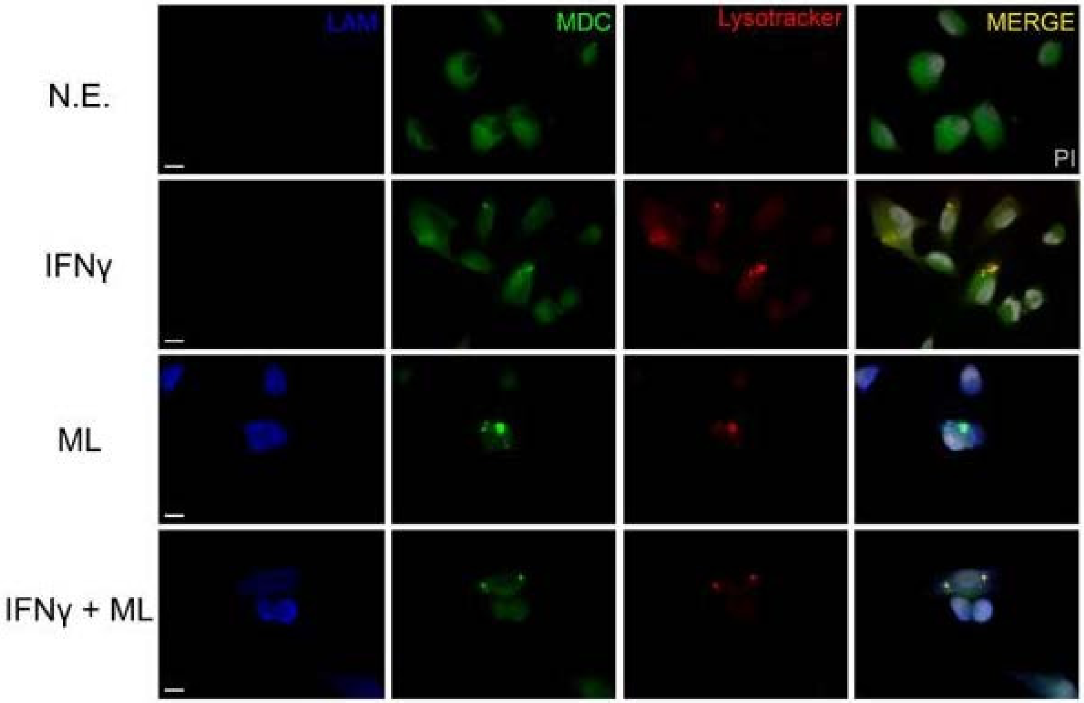

Supplementary Figure S1. Interferon (IFN)γ induces autophagic flux in ST88-14 Schwann cells. Schwann cells from the ST88-14 line were infected with M. leprae (ML) killed at a concentration of 50:1 for 1 h and treated with IFNγ for 18 h at 37 °C. The cells were fixed and the occurrence of autophagy was assessed by immunofluorescence microscopy using monodansylcadaverine (MDC, green dots) staining for autophagic vacuoles, lysosomes were stained with Lysotracker (red), nuclei with propidium iodide (PI, gray), and the ML by labeling with anti-LAM (blue). The figures are representative of one experiment. NE: not stimulated.
